# Supplementary material for: Changes in tobacco sales before, during, and after the COVID-19 pandemic in Japan: An interrupted time series analysis
Source: PLoS One. 2026 Jun 5;21(6):e0350820. doi: 10.1371/journal.pone.0350820 (PMC13240920; doi:10.1371/journal.pone.0350820)
Supplement: Attachment — Submitted filename: Response to Reviewers_C19Smoking-07.docx [file pone.0350820.s002.docx]

**Reviewer #1**: The authors used several data sources to provide evidence on changes in cigarette sales and smoking in Japan before and after the COVID-19 pandemic. The authors found increases in cigarette sales and purchase after the pandemic compared to the pre-pandemic levels and trends.

There are some major and minor concerns (not listed in order) that need to be considered.

Dear Reviewer 1,

Thank you very much for your careful review and thoughtful comments on our manuscript. We sincerely appreciate the time and effort you devoted to evaluating our work. Your comments were extremely helpful and led us to improve the manuscript substantially.

In the process of revision, additional analyses became necessary in response to the reviewers’ comments. During this period, newer data also became available. Accordingly, we updated the data sets and re-conducted the analyses using the most recent data available.

Specifically, for the number of cigarette sales, the study period was updated from the first quarter of 2015 to the first quarter of 2025 to the first quarter of 2015 to the fourth quarter of 2025. For National tobacco expenditures, the study period was updated from January 2015 to May 2025 to January 2015 to February 2026.

These updates are reflected in the revised manuscript and in our point-by-point response. We are very grateful for your valuable comments, which have helped us strengthen the manuscript.

1. Can you explain the data clearly, providing detailed information on what is available and how you used it in your analysis?

Thank you for this helpful comment. The Data sources section was revised to clarify what information was available from each dataset and how it was used in the present study. Specifically, the revised text now states that the cigarette sales analysis used the quarterly number of cigarettes sold, that the household expenditure analysis used price-adjusted monthly tobacco expenditures for households with two or more members, and that smoking prevalence data were annual percentages and therefore were summarized descriptively rather than analyzed in the interrupted time-series model.

The following sentences were added to the Data sources section of the revised manuscript:

New (P3, L95) In this study, the quarterly number of cigarettes sold was used for the analysis of cigarette sales trends.

New (P4L, 106) Thus, the monthly analysis was based on price-adjusted tobacco expenditures for households with two or more members.

New (P4L, 113) Because these data were available as annual national percentages, smoking prevalence in men and women was summarized descriptively rather than analyzed using the interrupted time-series model.

2. Model specification: I am not sure about the controls in the model, but the model should account for seasonality. For example, quarterly or monthly FE should be included in the models to account for seasonality. I suggest writing a formal model and defining terms.

Thank you for this helpful comment. We agree that autocorrelation and seasonality should be handled explicitly in the present time-series analyses. In response, we revised the analytic approach to account for seasonality using quarter indicators for the quarterly sales data and month indicators for the monthly expenditure data, and to account for autocorrelation using linear mixed models with an AR(1) covariance structure.

The following sentences were added.

New (P4, L133): For quarterly cigarette sales, seasonality was modeled using quarter indicators (Q2–Q4), with Q1 as the reference category. For monthly tobacco expenditures, seasonality was modeled using month indicators (February–December), with January as the reference category. To account for autocorrelation, the models were fitted using linear mixed models with an AR(1) covariance structure.

The results tables have been revised as follows.

Table 1. Parameter estimates from the interrupted time-series segmented regression model for quarterly cigarette sales adjusted for seasonality and autocorrelation.

|  |  |  | 95% confidence interval | |  |
| --- | --- | --- | --- | --- | --- |
|  |  | Estimate | Lower limit | Upper limit | P-value |
| Intercept |  | 244.982 | 228.278 | 261.685 | <0.001 |
| Baseline trend |  | -9.991 | -11.214 | -8.767 | <0.001 |
| After the first state of emergency declaration | Level change | -17.468 | -41.080 | 6.143 | 0.133 |
|  | Slope change | 7.962 | 4.858 | 11.067 | <0.001 |
| After COVID-19 was downgraded to a common infectious disease | Level change | -1.481 | -29.119 | 26.157 | 0.910 |
|  | Slope change | -0.924 | -5.227 | 3.379 | 0.643 |
| Q2 |  | 22.254 | 8.958 | 35.551 | 0.002 |
| Q3 |  | 42.881 | 29.759 | 56.002 | <0.001 |
| Q4 |  | 19.110 | 5.973 | 32.248 | 0.006 |

Note: Seasonality was modeled using quarter indicators (Q2–Q4), with Q1 as the reference category. Autocorrelation was accounted for using a linear mixed model with an AR(1) covariance structure. P-values were obtained from t-tests for fixed-effect parameters in the linear mixed model, using the denominator degrees-of-freedom method specified in SPSS. Values <0.05 were considered statistically significant.

Table 2. Parameter estimates from the interrupted time-series segmented regression model for monthly tobacco expenditures adjusted for seasonality and autocorrelation.

|  |  |  | 95% confidence interval | |  |
| --- | --- | --- | --- | --- | --- |
|  |  | Estimate | Lower limit | Upper limit | P-value |
| Intercept |  | 797.883 | 724.578 | 871.187 | <0.001 |
| Baseline trend |  | -2.501 | -4.037 | -0.964 | 0.002 |
| After the first state of emergency declaration | Level change | 132.004 | 42.281 | 221.727 | 0.005 |
|  | Slope change | -1.294 | -5.039 | 2.451 | 0.486 |
| After COVID-19 was downgraded to a common infectious disease | Level change | -17.578 | -120.702 | 85.546 | 0.731 |
|  | Slope change | 5.892 | 0.731 | 11.053 | 0.027 |
| February |  | -51.703 | -115.314 | 11.908 | 0.110 |
| March |  | 30.380 | -41.112 | 101.873 | 0.402 |
| April |  | 3.547 | -69.484 | 76.577 | 0.923 |
| May |  | 45.424 | -28.406 | 119.255 | 0.225 |
| June |  | 46.667 | -27.042 | 120.376 | 0.212 |
| July |  | 75.578 | 2.017 | 149.140 | 0.044 |
| August |  | 60.454 | -12.967 | 133.875 | 0.106 |
| September |  | 133.170 | 59.912 | 206.429 | <0.001 |
| October |  | -45.150 | -118.046 | 27.747 | 0.222 |
| November |  | -42.058 | -113.603 | 29.487 | 0.247 |
| December |  | 9.502 | -55.873 | 74.877 | 0.774 |

Note: Seasonality was modeled using month indicators (February–December), with January as the reference category. Autocorrelation was accounted for using a linear mixed model with an AR(1) covariance structure. P-values were obtained from t-tests for fixed-effect parameters in the linear mixed model, using the denominator degrees-of-freedom method specified in SPSS. Values <0.05 were considered statistically significant.

The figures presenting the results have been revised as follows.

New: Fig 1. Trend in cigarette sales before and after the first state of emergency declaration.

Note: The solid line shows fitted values from the interrupted time-series segmented regression model adjusted for seasonality and autocorrelation. The dashed line shows the predicted number of cigarette sales had the COVID-19 pandemic and the first state of emergency not occurred, assuming continuation of the pre-intervention trend (counterfactual). The time variable was centered at 0 for January–March 2020; thus, the intercept represents the estimated cigarette sales in that quarter.

New: Fig 2. Trend in monthly tobacco expenditures per household (households with two or more members) before and after the first state of emergency declaration.

Note: The round markers indicate tobacco expenditures adjusted for price fluctuations based on cigarette prices from the Ministry of Internal Affairs and Communications’ Retail Price Statistics Survey. Price increases occurred because of base price adjustments in April 2016 and April 2017, tobacco tax increases in October 2018, October 2020, and October 2021, and a revision of the consumption tax rate in 2019. The solid line shows fitted values from the interrupted time-series segmented regression model adjusted for seasonality and autocorrelation. The dashed line shows the predicted monthly tobacco expenditures per household if the COVID-19 pandemic and the first state of emergency had not occurred, assuming continuation of the pre-intervention trend (counterfactual). The time variable was centered at 0 for March 2020; thus, the intercept represents the estimated monthly expenditure in March 2020.

The relevant text in the Results section has been revised as follows.

Old (P5, L118): Using a segmented regression ITS model with two intervention points (April–June 2020 and April–June 2023), the level change at the first intervention was not statistically significant, whereas the slope became significantly less negative after the first declaration (p = 0.002), indicating a deceleration in the declining trend. In the first quarter of 2020, cigarette sales showed little change compared to the previous quarter; however, the post-intervention trend corresponded to a smaller quarterly decline (approximately 300 million cigarettes) compared with the pre-intervention period.

Nwe (P5, L166): After adjustment for seasonality and autocorrelation, the level change at the first intervention was not statistically significant, whereas the slope became significantly less negative after the first declaration (p < 0.001), indicating a deceleration in the declining trend. In the first quarter of 2020, cigarette sales showed little change compared to the previous quarter; however, the post-intervention trend corresponded to a smaller quarterly decline (approximately 200 million cigarettes) compared with the pre-intervention period.

Nwe (P5, L174): The quarter indicators were statistically significant, supporting the presence of seasonal variation in cigarette sales.

Old (P6, L142): From January 2015 to March 2020, price-adjusted monthly tobacco expenditures exhibited a declining trend with periodic fluctuations, decreasing from approximately 1,000 JPY to 800 JPY (Fig 2, Table 2). In April 2020, when the government declared the first state of emergency, price-adjusted monthly tobacco expenditures rose sharply by 153 JPY compared to the previous month (p < 0.001). However, from that point through April 2023, the expenditures continued to decline; the change in slope after April 2020 was not statistically significant (p = 0.251), indicating no evidence that the rate of decline differed from the pre-pandemic trend. After May 2023, when COVID-19 was downgraded to a common infectious disease, the rate of decline in price-adjusted monthly tobacco expenditures slowed, although the change was not statistically significant.

Nwe (P6, L208): From January 2015 to March 2020, price-adjusted monthly tobacco expenditures exhibited a declining trend with periodic fluctuations, decreasing from approximately 1,000 JPY to 800 JPY (Fig 2, Table 2). After adjustment for seasonality and autocorrelation, in April 2020, when the government declared the first state of emergency, price-adjusted monthly tobacco expenditures increased significantly by 132 JPY compared to the previous month (p = 0.005). However, from that point through April 2023, the expenditures continued to decline; the change in slope after April 2020 was not statistically significant (p = 0.486), indicating no evidence that the rate of decline differed from the pre-pandemic trend. After May 2023, when COVID-19 was downgraded to a common infectious disease, the rate of decline in price-adjusted monthly tobacco expenditures became significantly less steep (p = 0.027), whereas the level change was not statistically significant (p = 0.731).

Several month indicators were also statistically significant, supporting the presence of seasonal variation in tobacco expenditures.

3. Lines 104 to 106: Cite methodological studies that presented this approach as valid and how they are interpreted. Here is one of such studies: <https://journals.sagepub.com/doi/abs/10.1177/00131640021970358>

Thank you for this helpful comment. We revised the *Statistical methods* section to clarify the interpretation of the segmented regression coefficients. Specifically, we now state explicitly that the level-change parameter represents the immediate change at the first post-intervention time point relative to the value expected from the trend in the immediately preceding period, whereas the slope-change parameter represents the change in trend after the intervention relative to that preceding period. We also added the methodological reference by Huitema and McKean to support this clarification.

The following sentences and reference were changed and added at the Statistical methods.

New (P5, L149)

Following previous methodological guidance on segmented regression design specification [22], the level-change parameter for each intervention was defined as the immediate deviation at the first post-intervention time point from the value expected under continuation of the trend in the immediately preceding period, whereas the slope-change parameter was defined as the change in trend after the intervention relative to that immediately preceding period.

22. Huitema BE, McKean JW. Design specification issues in time-series intervention models. Educ Psychol Meas. 2000;60(1):38-58. https://doi.org/10.1177/0013164002197035

4. From Figure 1 and the results reported in Table 1, it is clear that the increase was not temporary. The effect of the pandemic on cigarette sales is a permanent change in the rate of decline, which lasted through 2024. I am not sure about how this was described as temporary. That change in slope never went away throughout the sample period. In Figure 2, we see a permanent shift in the level after the onset of the pandemic. That gap between the expected (counterfactual) and actual trends, although diminishing over time, persisted and even widened after May 2023.

Thank you for this important comment. The term “temporary” was imprecise and has been revised. Specifically, the description of cigarette sales was modified to avoid implying a temporary increase and to better reflect the sustained deceleration in the declining trend after the onset of the pandemic. In addition, the description of household tobacco expenditures was revised from “temporary increase” to “immediate increase in the level” in April 2020, which is more consistent with Figure 2 and Table 2.

The following sentences were changed to the Discussion section.

Old (P7, L192): However, the observed slowdown in the declining trend may suggest changes in purchasing and use patterns, including stockpiling or a temporary increase in smoking intensity among continuing smokers, similar to patterns reported in the United States.

New (P9, L306): However, the observed slowdown in the declining trend may suggest a sustained change in purchasing and use patterns after the onset of the pandemic, including stockpiling or changes in smoking intensity among continuing smokers, similar to patterns reported in the United States.

Old (P8, L227): In our study, the slowdown in the declining trend of cigarette sales and the temporary increase in price-adjusted tobacco expenditures immediately after April 2020 may reflect changes in purchasing and use patterns, such as stockpiling and/or a short-term increase in smoking intensity among continuing smokers; however, these data cannot distinguish between these explanations.

New (P10, L364): In our study, the slowdown in the declining trend of cigarette sales and the immediate increase in the level of price-adjusted tobacco expenditures in April 2020 may reflect changes in tobacco purchasing patterns, such as stockpiling, and possibly changes in smoking intensity among continuing smokers; however, these data cannot distinguish between these explanations.

Old (P10, L309): However, they did not result in a substantial reduction in smoking prevalence and were more consistent with a shift in purchasing and smoking patterns among continuing smokers—such as temporary stockpiling and/or a modest increase in smoking intensity—than with a meaningful population-level decline in smoking behavior.

New (P13, L537): However, they did not result in a substantial reduction in smoking prevalence and were more consistent with a shift in tobacco purchasing patterns, including stockpiling, and possible changes in smoking intensity among continuing smokers than with a meaningful population-level decline in smoking behavior.

5. Lines 205 – 207: Should be in the intro before setting up the hypothesis.

Thank you for this helpful suggestion. The description of the characteristics of Japan’s first state of emergency and its potential relevance to smoking-related behaviors was moved from the Discussion to the Introduction, before the study aim, to improve the logical flow of the manuscript. In the Introduction, the wording was also revised slightly to make the statement more appropriate as background information rather than as interpretation of the study findings.

Old (P8, L205): Japan’s first state of emergency did not involve a full lockdown, such as a citywide blockade or mandatory curfew, but instead included limitations on outdoor activities and reduced interpersonal contact to the extent feasible. Overall, these pandemic-related changes in daily routines and coping behaviors may have reduced the likelihood of continued declines in smoking-related behaviors during the restriction period.

New (P3, L83): Japan’s first state of emergency did not involve a full lockdown, such as a citywide blockade or mandatory curfew, but instead included requests to limit outdoor activities and reduce interpersonal contact where feasible. Such pandemic-related changes in daily routines and coping behaviors may have influenced smoking-related behaviors during the restriction period.

6. Line 217: Again, I don't know why the authors claim there was a temporary increase. You moved up to a different intercept and continued with the same trend. Levels never shifted to the original, so how is that temporary? It was a rather permanent increase in the level.

Thank you for this important comment. The phrase “(P8, L227) temporary increase” was revised because it did not accurately reflect the segmented regression results. The revised text now describes the expenditure finding as an immediate increase in the level of price-adjusted tobacco expenditures in April 2020. The related wording was also revised to be more cautious in its interpretation.

The following sentences were changed to the Discussion section.

Old (P8, L227): In our study, the slowdown in the declining trend of cigarette sales and the temporary increase in price-adjusted tobacco expenditures immediately after April 2020 may reflect changes in purchasing and use patterns, such as stockpiling and/or a short-term increase in smoking intensity among continuing smokers; however, these data cannot distinguish between these explanations.

New (P10, L364): In our study, the slowdown in the declining trend of cigarette sales and the immediate increase in the level of price-adjusted tobacco expenditures in April 2020 may reflect changes in tobacco purchasing patterns, such as stockpiling, and possibly changes in smoking intensity among continuing smokers; however, these data cannot distinguish between these explanations.

7. The figure title and captions in the text are more confusing and make reading difficult to distinguish. Add “Note:” to figure captions.

Thank you for this helpful comment. The figure titles and captions were revised to improve clarity and readability. In particular, the wording of the captions was simplified and organized so that the content of each figure can be distinguished more easily. In addition, “Note:” was added to the figure captions as suggested.

Old : Fig 1. Trend in cigarette sales before and after the first state of emergency declaration.

The solid line represents fitted values from the segmented regression ITS model. The dashed line represents the predicted number of cigarette sales had the COVID-19 pandemic and the first state of emergency not occurred, assuming continuation of the pre-intervention trend (counterfactual). The time variable was centered at 0 for January–March 2020; thus, the model intercept corresponds to the estimated cigarette sales in that quarter.

New: Fig 1. Trend in cigarette sales before and after the first state of emergency declaration.

Note: The solid line shows fitted values from the interrupted time-series segmented regression model adjusted for seasonality and autocorrelation. The dashed line shows the predicted number of cigarette sales had the COVID-19 pandemic and the first state of emergency not occurred, assuming continuation of the pre-intervention trend (counterfactual). The time variable was centered at 0 for January–March 2020; thus, the intercept represents the estimated cigarette sales in that quarter.

Old: Fig 2. Trend in monthly tobacco expenditures per household (households with two or more members) before and after the first state of emergency declaration.

The round markers indicate tobacco expenditure amounts adjusted for price fluctuations based on cigarette prices from the Ministry of Internal Affairs and Communications’ Retail Price Statistics Survey. Price increases occurred due to base price adjustments in April 2016 and April 2017; tobacco tax increases in October 2018, October 2020, and October 2021; and a revision of the consumption tax rate in 2019. The solid line represents fitted values from the segmented regression ITS model. The dashed line represents the predicted monthly tobacco expenditures per household if the COVID-19 pandemic and the first state of emergency had not occurred, assuming continuation of the pre-intervention trend (counterfactual). The time variable was centered at 0 for March 2020; thus, the model intercept corresponds to the estimated monthly expenditure in March 2020.

New: Fig 2. Trend in monthly tobacco expenditures per household (households with two or more members) before and after the first state of emergency declaration.
Note: The round markers indicate tobacco expenditures adjusted for price fluctuations based on cigarette prices from the Ministry of Internal Affairs and Communications’ Retail Price Statistics Survey. Price increases occurred because of base price adjustments in April 2016 and April 2017, tobacco tax increases in October 2018, October 2020, and October 2021, and a revision of the consumption tax rate in 2019. The solid line shows fitted values from the interrupted time-series segmented regression model adjusted for seasonality and autocorrelation. The dashed line shows the predicted monthly tobacco expenditures per household if the COVID-19 pandemic and the first state of emergency had not occurred, assuming continuation of the pre-intervention trend (counterfactual). The time variable was centered at 0 for March 2020; thus, the intercept represents the estimated monthly expenditure in March 2020.

Fig 3. Trends in the rate of heavy smoking among individuals aged 40 years and older.

Heavy smokers are defined as individuals who have smoked more than 100 cigarettes in total or have smoked for over six months and reported smoking within the past month.

Fig 3. Trends in the prevalence of heavy smoking among individuals aged 40 years and older.
Note: Heavy smokers are defined as individuals who have smoked more than 100 cigarettes in total or for more than 6 months and who reported smoking within the past month.

In addition, to maintain consistency with the revised interpretation presented in the final part of the manuscript, the Abstract and Conclusion have been revised accordingly.

**Reviewer #2**: This paper uses segmented interrupted time series (ITS) models to analyze three national-level data sources (cigarette sales, household tobacco expenditures, and smoking prevalence) to examine how the COVID-19 pandemic affected smoking behavior in Japan. The authors report that the declining trend in cigarette sales decelerated after the first state of emergency (April 2020) and household tobacco expenditures spiked by approximately 19%, but smoking prevalence did not meaningfully change. The topic is policy-relevant, and the multi-data-source design is a strength. However, there are several concerns that should be addressed.

Dear Reviewer 2,

Thank you very much for your careful review and thoughtful comments on our manuscript. We sincerely appreciate the time and effort you devoted to evaluating our work. Your comments were extremely helpful and led us to improve the manuscript substantially.

In the process of revision, additional analyses became necessary in response to the reviewers’ comments. During this period, newer data also became available. Accordingly, we updated the data sets and re-conducted the analyses using the most recent data available.

Specifically, for the number of cigarette sales, the study period was updated from the first quarter of 2015 to the first quarter of 2025 to the first quarter of 2015 to the fourth quarter of 2025. For National tobacco expenditures, the study period was updated from January 2015 to May 2025 to January 2015 to February 2026.

These updates are reflected in the revised manuscript and in our point-by-point response. We are very grateful for your valuable comments, which have helped us strengthen the manuscript.

1. Autocorrelation and seasonality. Monthly and quarterly time-series data are expected to exhibit autocorrelation, and tobacco purchasing has well-documented seasonal patterns. Standard OLS-based segmented regression will produce incorrect standard errors and p-values if autocorrelation is not addressed. The manuscript mentions that only in the limitations section as a caveat, rather than having it tested or corrected. Seasonality was likewise not modeled. I recommend the authors account for autocorrelation and seasonality adjustments, and provide standard diagnostic tests (e.g., Durbin-Watson statistic, Ljung-Box test) for the residuals.

Thank you for this helpful comment. We agree that autocorrelation and seasonality should be handled explicitly in the present time-series analyses. In response, we revised the analytic approach to account for seasonality using quarter indicators for the quarterly sales data and month indicators for the monthly expenditure data, and to account for autocorrelation using linear mixed models with an AR(1) covariance structure. We also examined the Durbin–Watson statistic as a standard residual diagnostic in the corresponding ordinary least-squares segmented regression models.

The following sentences were added.

New (P4, L133): For quarterly cigarette sales, seasonality was modeled using quarter indicators (Q2–Q4), with Q1 as the reference category. For monthly tobacco expenditures, seasonality was modeled using month indicators (February–December), with January as the reference category. To account for autocorrelation, the models were fitted using linear mixed models with an AR(1) covariance structure. As a residual diagnostic, the Durbin–Watson statistic was examined in the corresponding ordinary least-squares segmented regression models.

The results tables have been revised as follows.

Table 1. Parameter estimates from the interrupted time-series segmented regression model for quarterly cigarette sales adjusted for seasonality and autocorrelation.

|  |  |  | 95% confidence interval | |  |
| --- | --- | --- | --- | --- | --- |
|  |  | Estimate | Lower limit | Upper limit | P-value |
| Intercept |  | 244.982 | 228.278 | 261.685 | <0.001 |
| Baseline trend |  | -9.991 | -11.214 | -8.767 | <0.001 |
| After the first state of emergency declaration | Level change | -17.468 | -41.080 | 6.143 | 0.133 |
|  | Slope change | 7.962 | 4.858 | 11.067 | <0.001 |
| After COVID-19 was downgraded to a common infectious disease | Level change | -1.481 | -29.119 | 26.157 | 0.910 |
|  | Slope change | -0.924 | -5.227 | 3.379 | 0.643 |
| Q2 |  | 22.254 | 8.958 | 35.551 | 0.002 |
| Q3 |  | 42.881 | 29.759 | 56.002 | <0.001 |
| Q4 |  | 19.110 | 5.973 | 32.248 | 0.006 |

Note: Seasonality was modeled using quarter indicators (Q2–Q4), with Q1 as the reference category. Autocorrelation was accounted for using a linear mixed model with an AR(1) covariance structure. P-values were obtained from t-tests for fixed-effect parameters in the linear mixed model, using the denominator degrees-of-freedom method specified in SPSS. Values <0.05 were considered statistically significant.

Table 2. Parameter estimates from the interrupted time-series segmented regression model for monthly tobacco expenditures adjusted for seasonality and autocorrelation.

|  |  |  | 95% confidence interval | |  |
| --- | --- | --- | --- | --- | --- |
|  |  | Estimate | Lower limit | Upper limit | P-value |
| Intercept |  | 797.883 | 724.578 | 871.187 | <0.001 |
| Baseline trend |  | -2.501 | -4.037 | -0.964 | 0.002 |
| After the first state of emergency declaration | Level change | 132.004 | 42.281 | 221.727 | 0.005 |
|  | Slope change | -1.294 | -5.039 | 2.451 | 0.486 |
| After COVID-19 was downgraded to a common infectious disease | Level change | -17.578 | -120.702 | 85.546 | 0.731 |
|  | Slope change | 5.892 | 0.731 | 11.053 | 0.027 |
| February |  | -51.703 | -115.314 | 11.908 | 0.110 |
| March |  | 30.380 | -41.112 | 101.873 | 0.402 |
| April |  | 3.547 | -69.484 | 76.577 | 0.923 |
| May |  | 45.424 | -28.406 | 119.255 | 0.225 |
| June |  | 46.667 | -27.042 | 120.376 | 0.212 |
| July |  | 75.578 | 2.017 | 149.140 | 0.044 |
| August |  | 60.454 | -12.967 | 133.875 | 0.106 |
| September |  | 133.170 | 59.912 | 206.429 | <0.001 |
| October |  | -45.150 | -118.046 | 27.747 | 0.222 |
| November |  | -42.058 | -113.603 | 29.487 | 0.247 |
| December |  | 9.502 | -55.873 | 74.877 | 0.774 |

Note: Seasonality was modeled using month indicators (February–December), with January as the reference category. Autocorrelation was accounted for using a linear mixed model with an AR(1) covariance structure. P-values were obtained from t-tests for fixed-effect parameters in the linear mixed model, using the denominator degrees-of-freedom method specified in SPSS. Values <0.05 were considered statistically significant.

The figures presenting the results have been revised as follows.

New: Fig 1. Trend in cigarette sales before and after the first state of emergency declaration.

Note: The solid line shows fitted values from the interrupted time-series segmented regression model adjusted for seasonality and autocorrelation. The dashed line shows the predicted number of cigarette sales had the COVID-19 pandemic and the first state of emergency not occurred, assuming continuation of the pre-intervention trend (counterfactual). The time variable was centered at 0 for January–March 2020; thus, the intercept represents the estimated cigarette sales in that quarter.

New: Fig 2. Trend in monthly tobacco expenditures per household (households with two or more members) before and after the first state of emergency declaration.

Note: The round markers indicate tobacco expenditures adjusted for price fluctuations based on cigarette prices from the Ministry of Internal Affairs and Communications’ Retail Price Statistics Survey. Price increases occurred because of base price adjustments in April 2016 and April 2017, tobacco tax increases in October 2018, October 2020, and October 2021, and a revision of the consumption tax rate in 2019. The solid line shows fitted values from the interrupted time-series segmented regression model adjusted for seasonality and autocorrelation. The dashed line shows the predicted monthly tobacco expenditures per household if the COVID-19 pandemic and the first state of emergency had not occurred, assuming continuation of the pre-intervention trend (counterfactual). The time variable was centered at 0 for March 2020; thus, the intercept represents the estimated monthly expenditure in March 2020.

The relevant text in the Results section has been revised as follows.

Old (P5, L118): Using a segmented regression ITS model with two intervention points (April–June 2020 and April–June 2023), the level change at the first intervention was not statistically significant, whereas the slope became significantly less negative after the first declaration (p = 0.002), indicating a deceleration in the declining trend. In the first quarter of 2020, cigarette sales showed little change compared to the previous quarter; however, the post-intervention trend corresponded to a smaller quarterly decline (approximately 300 million cigarettes) compared with the pre-intervention period.

Nwe (P5, L166): After adjustment for seasonality and autocorrelation, the level change at the first intervention was not statistically significant, whereas the slope became significantly less negative after the first declaration (p < 0.001), indicating a deceleration in the declining trend. In the first quarter of 2020, cigarette sales showed little change compared to the previous quarter; however, the post-intervention trend corresponded to a smaller quarterly decline (approximately 200 million cigarettes) compared with the pre-intervention period.

Nwe (P5, L174): The quarter indicators were statistically significant, supporting the presence of seasonal variation in cigarette sales. The Durbin–Watson statistic in the corresponding ordinary least-squares segmented regression model was 2.100.

Old (P6, L142): From January 2015 to March 2020, price-adjusted monthly tobacco expenditures exhibited a declining trend with periodic fluctuations, decreasing from approximately 1,000 JPY to 800 JPY (Fig 2, Table 2). In April 2020, when the government declared the first state of emergency, price-adjusted monthly tobacco expenditures rose sharply by 153 JPY compared to the previous month (p < 0.001). However, from that point through April 2023, the expenditures continued to decline; the change in slope after April 2020 was not statistically significant (p = 0.251), indicating no evidence that the rate of decline differed from the pre-pandemic trend. After May 2023, when COVID-19 was downgraded to a common infectious disease, the rate of decline in price-adjusted monthly tobacco expenditures slowed, although the change was not statistically significant.

Nwe (P6, L208): From January 2015 to March 2020, price-adjusted monthly tobacco expenditures exhibited a declining trend with periodic fluctuations, decreasing from approximately 1,000 JPY to 800 JPY (Fig 2, Table 2). After adjustment for seasonality and autocorrelation, in April 2020, when the government declared the first state of emergency, price-adjusted monthly tobacco expenditures increased significantly by 132 JPY compared to the previous month (p = 0.005). However, from that point through April 2023, the expenditures continued to decline; the change in slope after April 2020 was not statistically significant (p = 0.486), indicating no evidence that the rate of decline differed from the pre-pandemic trend. After May 2023, when COVID-19 was downgraded to a common infectious disease, the rate of decline in price-adjusted monthly tobacco expenditures became significantly less steep (p = 0.027), whereas the level change was not statistically significant (p = 0.731).

Several month indicators were also statistically significant, supporting the presence of seasonal variation in tobacco expenditures. The Durbin–Watson statistic in the corresponding ordinary least-squares segmented regression model was 1.669.

2. Absence of sensitivity analyses. There is no exploration of alternative model specifications, for example, different polynomial functions (now is all assumed linear). ITS results can be sensitive to polynomial choices.

Thank you for this important comment. We agree that interrupted time-series results can be sensitive to the specification of the underlying time trend. In response, we conducted sensitivity analyses by additionally fitting models that included a quadratic time term (time²) in the fixed-effects part of the model.

For quarterly cigarette sales, the quadratic term was not statistically significant, and the main findings were materially unchanged: the pre-intervention declining trend remained significant, the post-first-intervention slope change remained significantly positive, and the level and slope changes after the second intervention remained non-significant. These results suggest that the findings for cigarette sales were robust to this alternative specification.

For monthly tobacco expenditures, however, the quadratic term was statistically significant, and some of the intervention-related estimates changed. In particular, the immediate level change after the first intervention was attenuated and was no longer statistically significant, whereas the post-first-intervention slope change became statistically significant. These findings suggest that the monthly expenditure results were more sensitive to the specification of the underlying time trend and should therefore be interpreted with greater caution.

We have added these sensitivity analyses to the manuscript and summarized the results accordingly.

The following sentences were added.

New (P5, L153): In addition, as a sensitivity analysis, a quadratic time term (time²) was added to the fixed-effects part of the model to assess the robustness of the findings to possible nonlinearity in the underlying secular trend.

The results tables have been added as follows.

Table 3. Parameter estimates from the interrupted time-series segmented regression model for monthly tobacco expenditures including a quadratic time term, adjusted for seasonality and autocorrelation.

|  |  |  | 95% confidence interval | |  |
| --- | --- | --- | --- | --- | --- |
|  |  | Estimate | Lower limit | Upper limit | P-value |
| Intercept |  | 874.494 | 789.946 | 959.042 | <.001 |
| Baseline trend |  | 5.140 | -0.128 | 10.408 | 0.055 |
| Quadratic time term (time²) | | 0.12 | 0.04 | 0.199 | 0.004 |
| After the first state of emergency declaration | Level change | 76.389 | -12.434 | 165.211 | 0.090 |
|  | Slope change | -13.265 | -21.878 | -4.652 | 0.004 |
| After COVID-19 was downgraded to a common infectious disease | Level change | -27.931 | -120.688 | 64.827 | 0.545 |
|  | Slope change | -2.513 | -9.759 | 4.734 | 0.485 |
| February |  | -52.094 | -115.462 | 11.274 | 0.106 |
| March |  | 30.795 | -38.214 | 99.805 | 0.379 |
| April |  | 8.717 | -61.118 | 78.552 | 0.805 |
| May |  | 51.888 | -18.503 | 122.279 | 0.147 |
| June |  | 53.965 | -16.313 | 124.243 | 0.131 |
| July |  | 83.462 | 13.291 | 153.634 | 0.020 |
| August |  | 68.681 | -1.396 | 138.759 | 0.055 |
| September |  | 141.494 | 71.507 | 211.480 | <.001 |
| October |  | -37.003 | -106.837 | 32.831 | 0.296 |
| November |  | -34.492 | -103.681 | 34.698 | 0.325 |
| December |  | 15.440 | -49.707 | 80.587 | 0.639 |

Note: Seasonality was modeled using month indicators (February–December), with January as the reference category. Autocorrelation was accounted for using a linear mixed model with an AR(1) covariance structure. P-values were obtained from t-tests for fixed-effect parameters in the linear mixed model, using the denominator degrees-of-freedom method specified in SPSS. Values <0.05 were considered statistically significant.

The following sentences were added at Results.

New (P5, L177): As a sensitivity analysis, we additionally fitted a model including a quadratic time term (time²). For quarterly cigarette sales, the quadratic term was not statistically significant, and the direction and statistical significance of the intervention-related estimates were unchanged.

New (P7, L233): As a sensitivity analysis, we additionally fitted a model including a quadratic time term (time²). For monthly tobacco expenditures, the quadratic term was statistically significant (Table 3). In this model, the immediate level change after the first intervention was no longer statistically significant, whereas the post-first-intervention slope change became statistically significant.

The following sentences were changed and added at Discussion.

Old (P8, L230): Moreover, after May 2023, the change in the expenditure trend was not statistically significant, providing no clear evidence of a sustained shift in smoking-related purchasing at the population level.

New (P10, L367): Sensitivity analyses including a quadratic time term did not materially change the cigarette sales results, but the monthly tobacco expenditure results were less stable. In particular, for monthly tobacco expenditures, the immediate level change after the first intervention was no longer statistically significant when the quadratic term was included. Accordingly, the expenditure findings should be interpreted with greater caution than the cigarette sales findings. Moreover, after May 2023, the change in the expenditure trend was significant in the model adjusted for seasonality and autocorrelation, but this result was not robust in the quadratic sensitivity analysis, further supporting a cautious interpretation of the monthly tobacco expenditure series.

Old (P8L262): Although a deceleration in the declining trend of tobacco expenditures was observed after May 8, 2023, when COVID-19 was reclassified as a common infectious disease and legal restrictions on individual behaviors were lifted, this change was not statistically significant. The absence of a significant shift in tobacco expenditures may reflect Japan’s prolonged economic stagnation [41] and a cultural tendency to avoid abrupt behavioral change.

New (P11, L451): Although a deceleration in the declining trend of tobacco expenditures was observed after May 8, 2023, when COVID-19 was reclassified as a common infectious disease and legal restrictions on individual behaviors were lifted, this change should be interpreted cautiously because it was not robust in the quadratic sensitivity analysis. The absence of robust evidence for a clear shift in tobacco expenditures may reflect Japan’s prolonged economic stagnation [45] and a cultural tendency to avoid abrupt behavioral change.

The following sentences were changed and added at Limitations.

Old (P10, L299): Finally, as with interrupted time-series analyses in general, our interpretation assumes that no other major contemporaneous factors influenced the outcomes around the intervention points; residual confounding and modelling choices (e.g., specification of seasonality and autocorrelation) may have affected the estimates.

New (P13, L525): Finally, as with interrupted time-series analyses in general, our interpretation assumes that no other major contemporaneous factors influenced the outcomes around the intervention points; residual confounding and model specification choices may have affected the estimates. Sensitivity analyses using a quadratic time term suggested that the monthly tobacco expenditure results were less robust than the cigarette sales results.

3. Overstatement of conclusions relative to the data. The abstract and conclusion state that "smoking behavior among smokers appeared to intensify" and that these effects "persisted beyond May 2023." Neither claim is adequately supported by the data results. First, the sales/expenditure variables measure purchasing, not consumption. The authors themselves acknowledge that "these data cannot distinguish stockpiling from a true increase in cigarettes consumed per smoker," yet the abstract and conclusion reassert the intensification interpretation. Second, the "persistence" claim rests on a non-significant result at the second intervention point. This is a type II error concern: the non-significant estimate reflects absence of evidence, not evidence of persistence. I recommend the authors revise the abstract and conclusions to focus on changes in sales/purchasing patterns (even just to be more consistent with your title), with the “intensifying” and “persistence” interpretations discussed just as possibilities rather than conclusive findings.

Thank you for this important comment. We agree that our sales and expenditure measures capture purchasing rather than consumption, and that the previous wording was too strong. In response, we revised the Discussion to focus on changes in tobacco purchasing patterns and to describe changes in smoking intensity only as a possibility rather than a conclusion. The revised sentence now states that the findings may reflect tobacco purchasing patterns, such as stockpiling, and possibly changes in smoking intensity among continuing smokers, while also noting that the data cannot distinguish between these explanations.

The following sentences were changed and added in the Discussion section.

Old (P8, L227): In our study, the slowdown in the declining trend of cigarette sales and the temporary increase in price-adjusted tobacco expenditures immediately after April 2020 may reflect changes in purchasing and use patterns, such as stockpiling and/or a short-term increase in smoking intensity among continuing smokers; however, these data cannot distinguish between these explanations.

New (P10, L364): In our study, the slowdown in the declining trend of cigarette sales and the immediate increase in the level of price-adjusted tobacco expenditures in April 2020 may reflect changes in tobacco purchasing patterns, such as stockpiling, and possibly changes in smoking intensity among continuing smokers; however, these data cannot distinguish between these explanations.

4. The 153 JPY increase is translated to a ~19% rise, but the pre-treatment average of 800 JPY is calculated across all households with two or more members, even including non-smoking households. To aid interpretation, the authors should report (or estimate from published data) what fraction of Japanese households include a current smoker. This would allow readers to gauge the approximate per-smoker-household effect. Additionally, since it also excludes single-person households, the authors should note what proportion of Japanese households are single-person (e.g., approximately 38% as of the 2020 Population Census of Japan) and discuss how this exclusion may affect generalizability. Particularly, single-person households may have very different smoking patterns.

Thank you for this important comment. We agree that the household expenditure measure in our study represents the average expenditure across all households with two or more members, including households without current smokers, and therefore should not be interpreted directly as the change per smoking household. In response, we revised the Discussion and Limitations sections to clarify this point more explicitly. Using published Japanese household-based data, we additionally provided contextual interpretation of the expenditure change. Specifically, a recent study using the JMDC Claims Database reported that 25.47% of households were classified as smoking households before the policy period. Based on this figure, the observed 132 JPY increase in monthly tobacco expenditure corresponds approximately to 518 JPY per smoking household, assuming that the increase was concentrated among smoking households; similarly, the pre-intervention average of about 800 JPY corresponds approximately to 3,141 JPY per smoking household. We clarify in the revised manuscript that these values are only approximate and should be interpreted cautiously. We also added that, according to the 2020 Population Census, single-person households accounted for 38.1% of general households in Japan. Because the Family Income and Expenditure Survey excludes single-person households, we now state more explicitly that the expenditure findings may have limited generalizability to the overall household population.

The following sentences were changed and added in the Discussion section.

Old (P7, L198): Consistent with a short-term shift in purchasing, price-adjusted monthly tobacco expenditures per household increased by 153 JPY in April 2020. Because this measure represents a population-average monthly amount per household with two or more members, including non-smoking households, and reflects purchasing rather than direct consumption, the 153 JPY increase should be interpreted as modest in absolute terms, even though it corresponds to roughly a 19% rise relative to the pre-intervention level of about 800 JPY. These aggregate data cannot distinguish stockpiling from a true increase in cigarettes consumed per smoker.

New (P9, L313): Consistent with a short-term shift in purchasing, price-adjusted monthly tobacco expenditures per household increased by 132 JPY in April 2020. Because this measure represents the average monthly expenditure across all households with two or more members, including households without current smokers, and reflects purchasing rather than direct consumption, the 132 JPY increase should be interpreted as modest in absolute terms, even though it corresponds to approximately a 17% increase relative to the pre-intervention level of about 800 JPY. This estimate should not be interpreted directly as the change per smoking household, because the denominator includes all households with two or more members, irrespective of smoking status. Using published Japanese household-based data indicating that 25.47% of households were classified as smoking households [24], the observed increase corresponds approximately to 518 JPY per smoking household, and the pre-intervention average corresponds approximately to 3,141 JPY per smoking household; however, these are only rough approximations and should be interpreted cautiously. These aggregate data cannot distinguish stockpiling from a true increase in cigarettes consumed per smoker. Moreover, because the Family Income and Expenditure Survey excludes single-person households, the findings may have limited generalizability to the overall household population. According to the 2020 Population Census, single-person households accounted for 38.1% of general households in Japan [25], indicating that a substantial segment of household types was not represented in the expenditure analysis. Because tobacco purchasing and smoking patterns may differ in single-person households, the direction and magnitude of any resulting bias cannot be determined a priori.

The following references were added.

24. Tang MC, Wang M, Yin T. Indoor Smoking Bans and Children’s Health Outcomes in Japan. RIETI Discussion Paper Series 25-E-122. Available: https://www.rieti.go.jp/jp/publications/dp/25e122.pdf

25. Statistics Bureau, Ministry of Internal Affairs and Communications. Population and Households of Japan 2020: Overview of the Results of the 2020 Population Census. Available: https://www.stat.go.jp/english/data/kokusei/2020/summary/pdf/all.pdf

The following sentences were changed and added in the Limitation section.

Old (P10, L292): Second, sales and expenditure data reflect purchasing rather than actual consumption; therefore, the observed increase in April 2020 may partly represent stockpiling or shifts in purchasing timing. In addition, the Family Income and Expenditure Survey covers households with two or more members, which may limit generalisability to single-person households.

New (P12, L493): Second, sales and expenditure data reflect purchasing rather than actual consumption; therefore, the observed increase in April 2020 may partly reflect stockpiling or shifts in purchasing timing. In addition, the Family Income and Expenditure Survey covers only households with two or more members, and the expenditure estimates represent averages across all such households, including those without current smokers; accordingly, they should not be interpreted as direct estimates of change per smoking household. Published household-based data suggest that smoking households account for only a minority of Japanese households, and thus the implied increase per smoking household would be substantially larger than the all-household average, although such calculations are approximate. This may limit generalizability to single-person households, which accounted for 38.1% of general households in Japan in the 2020 Population Census and may have different tobacco purchasing and smoking patterns. Because single-person households comprised a large proportion of households, the expenditure findings should be interpreted primarily as applying to households with two or more members rather than to the entire household population.

The following sentences were changed and added in the Conclusion section.

Old (P10, L305): During the first state of emergency in Japan, the declining trend in tobacco sales decelerated. Furthermore, household spending on tobacco increased by approximately 20% following the declaration.

New (P13, L532): During the first state of emergency in Japan, the declining trend in tobacco sales decelerated. Furthermore, household spending on tobacco increased by approximately 17% following the declaration.

5. The authors note that heated tobacco products (HTPs) are excluded from the analysis, though its revenue now represents approximately 80% of conventional cigarette revenue in Japan, a substantial market shift occurring over the study period. If smokers transitioned between cigarettes and HTPs during the pandemic, the observed changes in the cigarette sales/expenditure could be partially attributable to cross-product substitution. I know that the authors cite one Osaka survey showing low switching rates (2.2%), but this is limited evidence from a single city during a short window and cannot be generalized to the national level over a longer time period. This limitation should be discussed more prominently and explicitly acknowledged as a potential confounder in the ITS results, rather than treated as a secondary limitation.

Thank you for this important comment. We agree that the exclusion of heated tobacco products (HTPs) should be discussed more explicitly as a potential source of confounding in the interpretation of our interrupted time-series results. In response, we revised the manuscript to give this limitation greater prominence. Specifically, we now state more clearly that, because HTPs were not included in our sales and expenditure analyses, changes in tobacco purchasing patterns during the pandemic may partly reflect substitution between cigarettes and HTPs. We also revised the text to avoid overinterpreting the Osaka study, noting that its low switching rates were observed in a single city during a limited time period and therefore cannot be generalized to the national level over the full study period. In addition, we strengthened the Limitations section to state explicitly that cross-product substitution may have confounded the ITS estimates.

The following sentences were changed and added in the Discussion section.

Old (P8, L209): The present study was limited to cigarette sales. In recent years, sales of heated tobacco products (HTPs) have increased, and their retail revenue now accounts for approximately 80% of that generated by conventional cigarettes [22].A shift from cigarettes to HTPs could influence overall tobacco sales volume. However, one study conducted in Osaka, Japan, reported that during the state of emergency, only 2.2% of individuals switched from cigarettes to HTPs, while just 0.6% switched in the opposite direction [10]. Therefore, it is possible that few smokers changed their tobacco product type during the pandemic, and the impact of such changes on this study’s findings may be limited.

New (P10, L345): The present study was limited to cigarette sales. In recent years, sales of heated tobacco products (HTPs) have increased, and their retail revenue now accounts for approximately 80% of that generated by conventional cigarettes [26]. Accordingly, substitution between cigarettes and HTPs may have affected the observed changes in cigarette sales and household tobacco expenditures and should be considered a potential confounder in the interpretation of the ITS results. One study conducted in Osaka, Japan, reported that during the state of emergency, only 2.2% of individuals switched from cigarettes to HTPs, while 0.6% switched in the opposite direction [10]. However, because this evidence was obtained in a single city during a limited period, it cannot be generalized to the national level over the longer study period.

The following sentences were changed and added in the Limitation section.

Old (P10, L296): Third, our sales series did not include heated tobacco products or other nicotine products, and substitution across products could bias inferences based on cigarette sales alone.

New (P12, L505): Third, our sales series did not include heated tobacco products or other nicotine products, and substitution across products may have confounded inferences based on cigarette sales and household tobacco expenditures. Because the market share of HTPs increased substantially during the study period, this limitation should be considered when interpreting the ITS results.

6. The NDB Open Data include only adults aged 40+, excluding younger adults (i.e., 20–39), a demographic likely with very different tobacco use patterns and pandemic-related behavioral changes compared to older adults. In Japan, smoking prevalence among younger men has historically been higher than among older age groups, and younger adults may be more responsive to social and environmental changes. This limitation is mentioned only briefly; it should be more explicitly flagged when interpreting the prevalence trends, and the authors should discuss the direction in which this exclusion might bias their findings.

Thank you for this important comment. We agree that the restriction of the NDB Open Data to adults aged 40 years and older should be more clearly acknowledged when interpreting the smoking prevalence findings. In response, we revised the manuscript to emphasize that the prevalence results do not capture younger adults aged 20–39 years, whose tobacco use patterns and responses to pandemic-related social and environmental changes may differ from those of older adults. We also clarified that this age restriction may have biased the observed prevalence trends, because changes in smoking behavior among younger adults may not be reflected in the present data. Accordingly, we revised both the Discussion and Limitations sections to state more explicitly that the smoking prevalence findings should be interpreted as applying to adults aged 40 years and older rather than to the entire adult population.

The following sentences were changed and added in the Discussion section.

New (P12, L484): However, because the NDB Open Data cover only adults aged 40 years and older, these findings should be interpreted cautiously and should not be generalized directly to younger adults aged 20–39 years. If younger adults were more responsive to pandemic-related social and environmental changes, the present data may have underestimated changes in smoking prevalence in the overall adult population.

The following sentences were changed and added in the Limitation section.

New (P13, L523): Accordingly, the prevalence findings may not be generalizable to the entire adult population and may underestimate changes if smoking behavior was more responsive among younger adults during the pandemic.

In addition, to maintain consistency with the revised interpretation presented in the final part of the manuscript, the Abstract and Conclusion have been revised accordingly.
